# Based on Gene Expression Analysis: Low-Density Neutrophil Expression Is a Characteristic of the Fast Responders Treated With Guselkumab for Psoriasis

**DOI:** 10.3389/fimmu.2022.865875

**Published:** 2022-05-26

**Authors:** Jiajing Lu, Yu Wang, Ying Li, Xiaoyuan Zhong, Yu Gong, Yangfeng Ding, Ning Yu, Yuling Shi

**Affiliations:** ^1^ Department of Dermatology, Shanghai Skin Disease Hospital, School of Medicine, Tongji University, Shanghai, China; ^2^ Institute of Psoriasis, School of Medicine, Tongji University, Shanghai, China

**Keywords:** psoriasis, guselkumab, RNA-seq, biomarkers, neutrophils

## Abstract

Psoriasis is a worldwide chronic inflammatory skin disease. The treatment of disease is usually designed according to its severity. In this research, RNA-seq was performed on 37 patients with psoriasis treated with guselkumab before and after treatment, and the patients were divided into fast responder and slow responder according to PASI score to analyze the differentially expressed genes (DEGs) between them. Moreover, The biological mechanism of psoriasis was explored by Kyoto Encyclopedia of Genes and Genomes (KEGG) analysis, Gene Ontology (GO) analysis, and Gene Set Enrichment Analysis (GSEA) analysis. And then, this protein-protein interaction network was constructed and 17 DEGs including IL-1β, CXCL8, S100A12 and MMP9 were analyzed by GSVA. DEGs were detected by GO and KEGG analysis of target genes, which were primarily associated with immune response, neutrophil activation, neutrophil degranulation. GSEA reminded that fast responders were mainly involved in low-density neutrophils and abundant NK cells. And the GSVA showed that the DEGs were down-regulated after the early stage of the fast responder and the reverse in the slow responder by GSVA analysis. On the whole, these results indicated that these DEGs may serve as a psoriasis potential diagnostic and predictive biomarkers after been treated by guselkumab.

## Introduction

Psoriasis is a chronic inflammatory skin disease characterized by scaly, inducible, erythematous plaques. The quality of life in patients can be with severe shadows. Neutrophil accumulation in the skin is a hallmark of psoriasis (1–3). The pathogenesis of psoriasis has been considered to be a combination of immune disorders, psoriasis-related susceptibility sites, psoriasis autoantigens, and a variety of environmental factors (1). Recent studies have shown that dendritic cells and other antigen-presenting cells (APCs) produce IL-23, which induces the differentiation and proliferation of CD4+ helper T lymphocytes–Th17 cells. The differentiated and mature Th17 cells can secrete IL-17, IL-21, IL-22 and other Th17 cytokines stimulate the excessive proliferation of keratinocytes. During this process, a large number of chemokines will also be released, which synergistically accelerate the inflammatory response. Therefore, Th17 cells and the IL-23/IL-17 axis may play a key role in the pathogenesis of psoriasis and become new therapeutic targets. The traditional treatments (glucocorticoids,vitamin D analogues, and phototherapy, etc.) are not very effective. With the development of the pathogenesis of psoriasis, the role of IL-17, IL-23, CXCL16, CXCL17 and tumor necrosis factor-α (TNF-α) in the immunoinflammatory process of psoriasis is becoming clear (4–6). The development of biological agents targeting these key cytokines has significantly improved the therapeutic effect and safety of psoriasis.

Guselkumab offers new hope for the treatment of psoriasis patients. It is an investigational human mAb specific for IL-23 (7–9). Guselkumab is a human IgG1λ monoclonal antibody (mAb) that binds selectively to the interleukin 23 (IL-23) protein with high specificity and affinity. IL-23, a regulatory cytokine, affects the differentiation, expansion, and survival of T cell subsets, (e.g., Th17 cells and Tc17 cells) and innate immune cell subsets, which represent sources of effector cytokines, including IL-17A, IL-17F and IL-22 that drive inflammatory disease. In humans, selective blockade of IL-23 was shown to normalize production of these cytokines. Levels of IL-23 are elevated in the skin of patients with plaque psoriasis. In *In vitro* models, guselkumab was shown to inhibit the bioactivity of IL-23 by blocking its interaction with cell surface IL-23 receptor, disrupting IL-23-mediated signaling, activation and cytokine cascades. Although relevant studies have been reported on its efficacy and safety internationally, there is still a lack of data on the Chinese psoriasis population.

In recent years, new insights into the molecular mechanisms and therapeutic targets of psoriasis have been provided by bioinformatics analysis (10). A pooled analysis of DEGs in psoriasis will remind us of the etiology of the disease, as these DEGs may reflect the common skin reactions that occur in many skin diseases in the absence of common causal mechanisms, and prognostic analyses have no evidence of significant differences between patients treated with guselkumab (11, 12).

In this study, we not only assessed the safety and clinical response of guselkumab in patients with psoriasis, but also divided patients into fast responders and slow responders based on their PASI score. We analyzed differentially expressed genes (DEGs) between the two groups by RNA-seq meta-analysis, and the relationship between fast and slow responder differential genes using GO and KEGG, and constructed a PPI network to discover potential psoriasis Prognostic biomarkers to provide reference for clinical treatment (13, 14).

## Materials and Methods

### Samples and Ethics

Samples were collected from patients with psoriasis (n=37) who were admitted to the Affiliated Dermatology Hospital of Tongji University between March 2020 and February 2021, including moderate to severe. Among them, patients receiving initial treatment with biological agents (n=21) and non-initially treatment with biological agents (n=16). This experiment was approved by the Research Ethics Committee of Tongji University Affiliated Dermatology Hospital. PASI score was (15.21 ± 2.39), PGA score was (3.66 ± 0.53), BSA score was (17.49 ± 4.50)% and DLQI score was (19.13 ± 3.88) among the psoriasis patients. During the treatment, 4 patients suspended the course of treatment for personal reasons, and the rest patients continued the treatment according to the normal course of treatment. 37 patients completed the 4-week treatment, 35 completed 8 weeks, 25 completed 16 weeks, 18 completed 24 weeks, and 5 completed all 48 weeks.

### Inclusion Criteria

① Subjects: patients aged ≥ 18 years, Clinical diagnosis of moderate to severe plaque Psoriasis over 6 months, PASI score ≥ 10 points, Physician Global Assessment (PGA) score ≥ 3 points, and BSA ≥ 10%. Nationality, race, gender, etc. ② Intervention measures: The experimental group received 100mg guselkumab at week 0 and week 4, and 100mg every 8 weeks thereafter. ③ Outcome indicators: a. Patients receiving initial treatment with biological agents with PASI score improvement rate of 75% (PASI 75) at 4 weeks were fast responders; others were low responders; b. The number of PASI 75 and PASI 90 for all patients at 48 weeks; c. Changes in PGA, skin disease Quality of Life Index (DLQI) scores and BSA.

### Exclusion Criteria

A. Subjects were mild plaque psoriasis or other types of psoriasis; B. The subjects were patients with psoriasis who did not respond to other biological agents; C. The study data were missing or there were no relevant outcome indicators.

### RNA Isolation and Library Preparation

TRIzol tries to extract total RNA with reagent according to the instructions. NanoDrop 2000 spectrophotometry(Thermo Scientific, USA) was used to measure and evaluate RNA purity and quantification. The Agilent 2100 bioanalyzer(Agilent Technologies, Santa Clara, CA, USA) was used to assess RNA integrity. Then the truseq mRNA LT sample was used to prepare the kit in accordance to the instructions. OE Biotechnology Ltd. (Shanghai, China)performed transcriptome sequencing and analysis.

### Serum Cytokine Analysis

Serum was collected from patients who has been received guselkumab before and at weeks 48 after treatment. The IL-17, IL-23, CXCL16 and CXCL17 levels were measured with R&D Systems kits(Minneapolis, Minn).

### RNA Sequencing and Differentially Expressed Genes Analysis

Illumina HiSeq X Ten platform was used to sequence the library and generate a 150 bp paired terminal sequence (1). Clean sequences of the human genome were mapped using HISAT2 (GRCh38) (2). Cufflinks was used to calculate the FPKM value of each gene, and htSEq-count was used to obtain the read count of each gene (1, 3, 4). DESeq (2012) R software package was used for differential expression analysis (5). Hierarchical cluster analysis of differentially expressed genes (DEGs) was used to reveal gene expression patterns in different groups and samples. Principal component analysis (PCA) is performed on DEG to determine orthogonal linear transformations of input data so that patterns of variation can be studied in a given data set.

### The Function and Pathway Enrichment of DEGs Were Analyzed

These DEGs are analyzed in cluster profilers to assign gene ontology terms for the roles that DEGs can play in biological processes, molecular function, and cell composition. The role of DEGs in various metabolic pathways uses the Gene Ontology (GO) and Kyoto Encyclopedia of Genes and Genomes (KEGG) as well as screening based approaches. Gene Set Enrichment analysis (GSEA) is a statistical method used to determine whether genes from a specific pathway or other predefined Gene sets are differentially expressed in different phenotypes (15). GSEA and clusterProfiler are used to analyze the Reactome path and define each functional cluster, and false detection rate (FDR) < 0.25, P < 0.05 was considered significant enrichment. The GSVA was used to execute the functional enrichment analysis to clarify the unique signaling pathways of variation trend of differentially expressed genes in fast responder and slow responder with psoriasis, and define the |correlation coefficient | > 0.5 cut-off value (16).

### PPI Network Analysis

PPI network analysis uses STRING to determine that STRING is a database that searches for protein interactions, including direct physical interactions between proteins and indirect functional correlations. CytoHubba as a useful addon in Cytoscape, provides 12 topological analysis methods which can be used to explore important nodes in biological networks. Molecular Complexity Detection (MCODE), which acts as a screening module for PPI networks, has a cut-off value of 10, node score cut-off value of 0.2, K‐core of 2 and a maximum depth of 100. GO analysis was performed for the first three modules using the STRING Enrichment plugin. FDR<0.05 was the cut-off standard for GO analysis.

## Results

### Guselkumab Improves the Clinical Manifestations of Psoriasis

**Figure 1:**
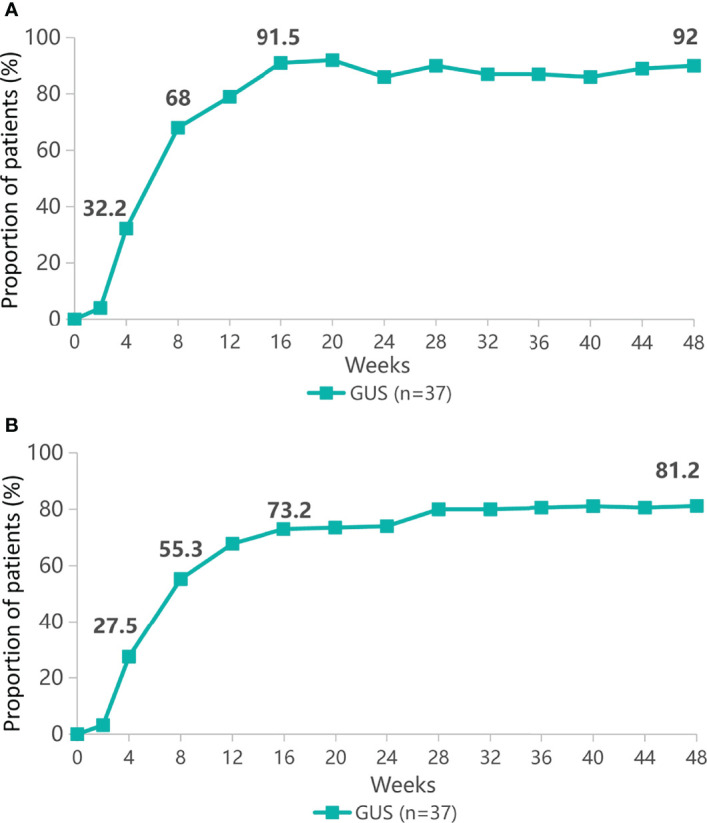
The proportion of patients with psoriasis area and severity index (PASI 75) remission **(A)** or PASI 90 remission **(B)** from baseline to 48 weeks.

**Figure 2:**
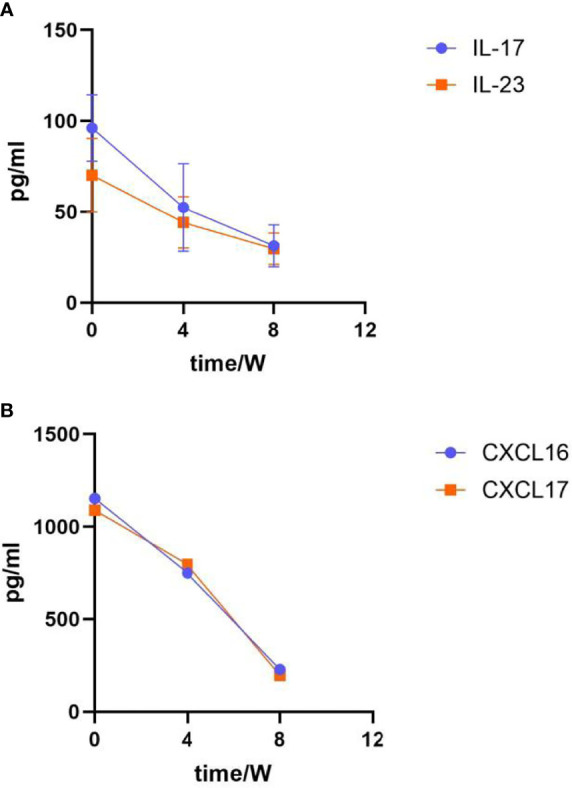
Laboratory indicators after guselkumab treatment. **(A)** Changes in IL-17 and IL-23 expression levels. The expression of IL-17 at baseline was (96.14 ± 18.35); Week 4 was (52.44 ± 24.12), Week 8 was (31.27 ± 11.54); The expression of IL-23 at baseline was (70.13 ± 20.26), Week 4 was (44.20 ± 14.19), Week 8 was (29.65 ± 8.64). **(B)** Changes in CXCL16 and CXCL17 expression level. The expression of CXCL16 at baseline was (1153.32 ± 25.53); Week 4 was (750.38 ± 15.37), Week 8 was (228.47 ± 11.43); The expression of CXCL17 at baseline was (1089.64 ± 18.17), Week 4 was (796.53 ± 20.63), Week 8 was (196.85 ± 7.98).

Patients with psoriasis (n=37) received a single subcutaneous injection of 100mg guselkumab. Clinical response was assessed up to week 48 on the basis of PASI response. Proportion of patients with PASI 75 in remission at week 48 was shown in **Figure 1A**. At week 4, PASI 75 response was achieved by 32.2% of patients. While at week 48, 92% of patients achieved PASI 75 response. The proportion of patients with PASI 90 is shown in **Figure 1B**, with PASI 90 in remission in 27.5% of patients at week 4 and 81.2% at week 48. The clinical laboratory indicators of psoriasis patients after treatment with guselkumab were shown in **Figure 2**. Guselkumab is generally considered to be well tolerated. As of week 48, no serious infection, malignancy, cardiovascular problems, severe hypersensitivity or death were reported.

### Preprocessing of Expression Data in Gene and Identification in DEGs

**Figure 3:**
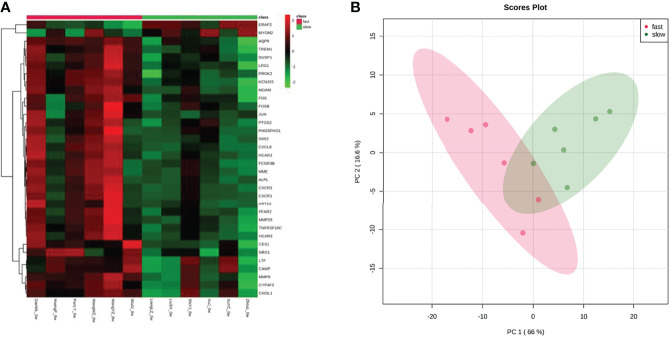
Assessment and visualization between fast responders and slow responders. **(A)** A heat map was generated from unsupervised clustering of differentially expressed transcripts between fast responders and slow responders. For clarity, the 79 transcripts with-1.5>log2(FC)>1.5, and p<0.05 were selected. Green indicates low expression levels whereas red indicates high expression levels. **(B)** PCA plots after the batch effect removal.

The gene expression matrices of fast responders and slow responders were normalized and the principal component analysis diagram was drawn. The results show that the normalized sample clustering is more obvious, which show that the source of samples is reliable (**Figure 3A**). Heatmap of DEGs indicated a significant difference between fast responders and slow responders with psoriasis (**Figure 3B**).

### The Function and Pathway Enrichment of DEGs Were Analyzed

**Figure 4:**
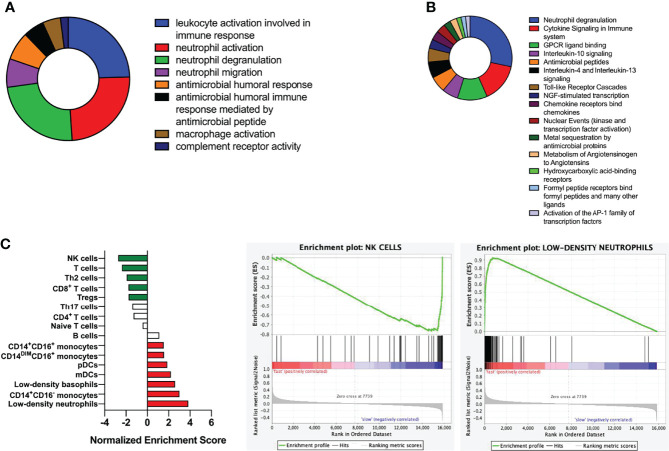
Functional and pathway enrichment analysis of DEGs. **(A)** Analysis of biological functions using GO enrichment. **(B)** Pathway analysis using KEGG. **(C)** Gene enrichment analysis; Kolmogorov-Smirnov test was used to calculate P-values.

Analysis of GO showed that DEGs was mainly related to Leukocyte activation and was involved in immune response, activation of neutrophils, degranulation of neutrophils, neutrophil migration and antimicrobial humoral response (**Figure 4A**). DEGs were mainly enriched in neutrophil degranulation, cytokine signaling in immune system and GPCR ligand binding, as demonstrated by KEGG analysis (**Figure 4B**). GSEA suggested fast responders with psoriasis was mainly involved in low-density neutrophils and abundant NK cells (**Figure 4C**).

### Correlation Analysis of the Seventeen Prognostic-Associated Genes

**Figure 5:**
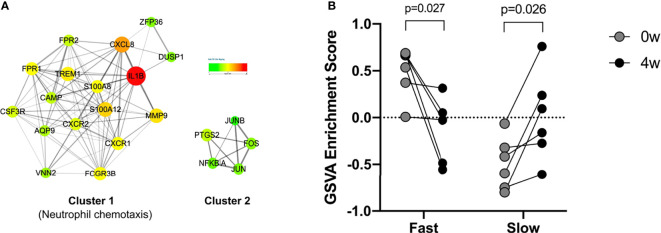
PPI network and GSVA analyses. **(A)** Protein-protein interaction (PPI) network of in fast responders with psoriasis patients. **(B)** GSVA analyses for the 17 genes.

The Upregulated DEG in the fast responders with psoriasis was constructed to form a PPI network, and formed two clusters. 17 genes in Cluster 1 were enriched in neutrophil chemotaxis (**Figure 5A**). The 17 genes were identified as marker genes, and GSVA was used to analyze their changes over time. The results showed that the response of the fast and slow groups was different. The genes of the fast responders were down-regulated after early treatment, while those of the slow responders were up-regulated after treatment (**Figure 5B**).

**Figure 6:**
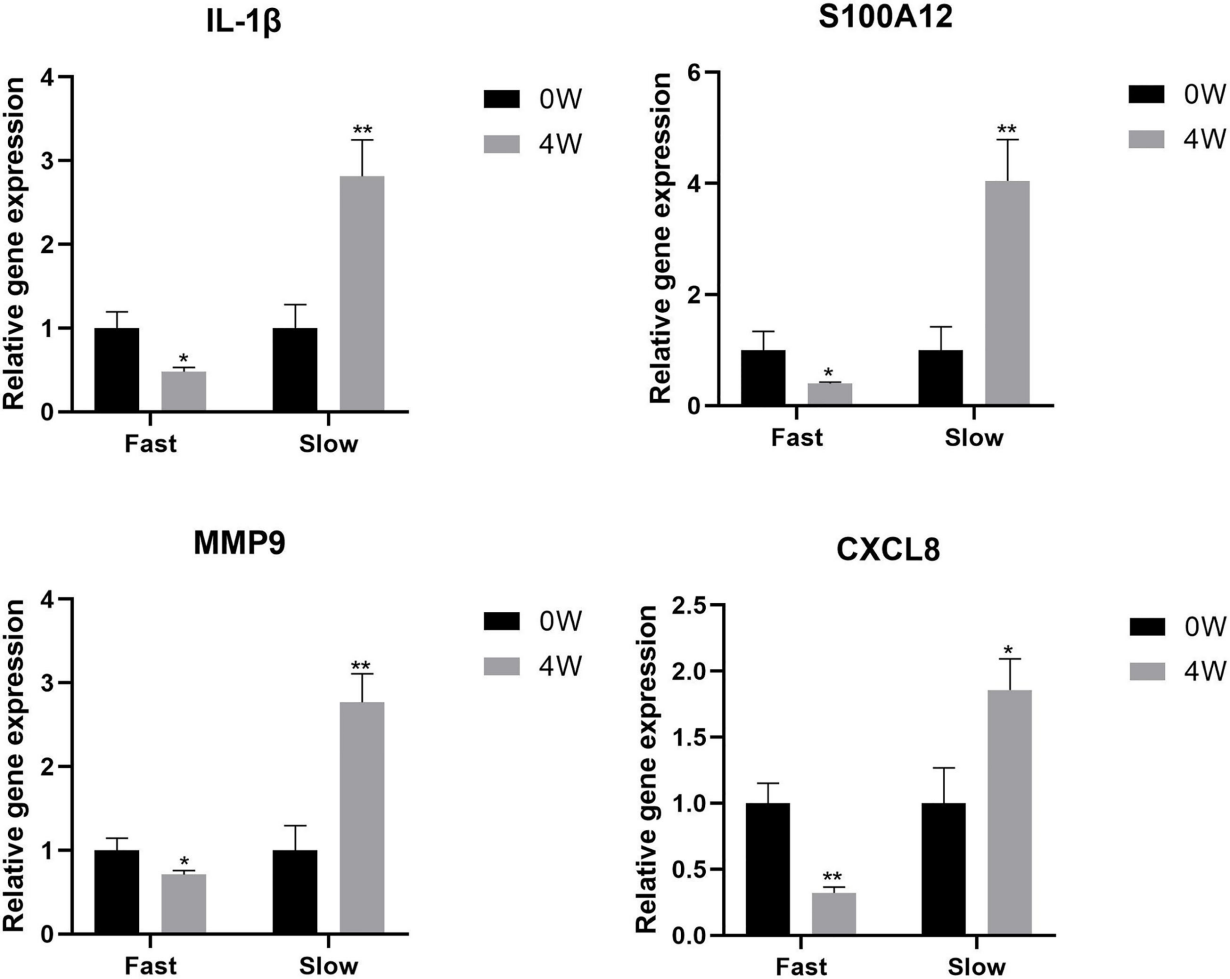
The expression level of IL-1β, S100A12, MMP9, and CXCL8 genes in 0W and 4W samples from fast responders and slow responders. *P < 0.05, **P < 0.01.

The expression of IL-1β, S100A12, MMP9, and CXCL8 genes was examined by qPCR in 0W and 4W samples from fast responders and slow responders. The expression level of these genes was down-regulated in fast responders after early treatment, while it showed the opposite trend in slow responders (*P<0.05, **P<0.01, **Figure 6**).

## Discussion

Psoriasis is a chronic inflammatory skin disease mediated by immunity that affects the quality of lives of patients significantly. So far, the detailed pathogenesis of psoriasis remains unclear. According to the current research, The interaction between immune cells and keratinocytes plays an important role in its pathogenesis. The pathway between cytokine and cytokine receptor is also very common in psoriasis, and the pathway of IL-23/IL-17 is the most studied currently. Many studies show that guselkumab as an important role in the pathway of IL-23/IL-17 in psoriasis. It suggests that it can play an important role in psoriasis. In recent studies, we used guselkumab to treat psoriasis with satisfactory results.

In our study, 37 patients with psoriasis were enrolled with guselkumab for a total course of 48 weeks, and almost all of them achieved ideal outcomes. DLQI, PGA, and BSA decreased significantly after 48 weeks of guselkumab treatment. PASI75 was achieved by 92% of patients and 81.2% achieved PASI90 at 48 weeks. This suggests that at 48 weeks, the psoriatic lesions was basically cleared in 81.2% of patients. IL17, IL-23, CXCL16 and CXCL17 was also significantly decreased by laboratory tests, suggesting that guselkumab has a good efficacy in the psoriasis treatment. CXCL16 is the second chemokine discovered so far that can exist in a membrane-bound form. CXCL16 expression levels are also elevated in some immune-mediated inflammatory diseases. Th1-type cytokines can promote the secretion of CXCL16, while Th2-type cytokines can inhibit the release of CXCL16 (17). Studies have shown that CXCL16 has chemotactic effects on CD4+ T cells, and further activates CD8+ T cells, thereby promoting the secretion of IFN-γ, IL-17 and TNF-α, etc., and is involved in the pathogenesis of psoriasis progress (18). CXCL17 is also a novel chemokine that is associated with the secretion of inflammatory factors such as IL-6 and TNF-α, etc. Therefore, they play an important role in the occurrence and development of psoriasis (19).

After that, we divided psoriasis patients into fast and slow responders using PASI 75, and analyzed the DEGs between fast-response and slow-response patients using biological information (17). Go analysis revealed that identified DEGs were enriched in leukocyte activation and involved in immune response, neutrophil activation, neutrophil degranulation, neutrophil migration and antimicrobial humoral response, and KEGG analysis has found that identified DEGs were mainly enriched in neutrophil degranulation, cytokine signaling in immune system and GPCR ligand binding. According to previous studies, the elevation of NK cell in the patients’ peripheral blood was not obvious, but they were recruited in the dermis. In our study, NK cell was found to be elevated in the fast responders of psoriasis, which was consistent with the previous results. We speculated that the recruitment of NK cells might be used as an indicator to evaluate the efficacy of guselkumab in clinical, but in the follow-up study, we will detect the recruitment of NK cells in peripheral blood and skin of patients. In addition, a PPI network was constructed and 17 genes was found, including IL-1β, CXCL8, S100A12 and MMP9 were down-regulated after the early stage of the fast responder and the reverse in the slow responder by GSVA analysis (18, 19). Some studies have suggested that the interaction between cytokine and cytokine, and the pathway of chemokine plays an important role in the psoriasis pathogenesis. In our analysis, we also found many cytokines interacting with guselkumab, and these cytokines were different from between fast and slow responders. Therefore, we believe that these cytokines can be used as predict patient response to guselkumab curative effect index, and in the later study, we will use skin lesions with peripheral blood of psoriasis patients to organize all experimental verification and to get more definitive conclusion.

Among these, The gene that changed the most is IL-1 β, the IL-1 family of cytokine also palys a key role in the pathogenesis of psoriasis. IL-1β plays an important role in the pathogenesis of psoriasis, IL-1β is known to be critical in IL-17-producing T cell differentiation and activation. Guselkumab inhibits the IL-23/T-assisted type 17 immune axis, which plays a key role in psoriasis. Although there have been a lot of researches, the mechanisms of IL-1β production in healthy and psoriatic skin, as well as the exact cellular and molecular mechanisms by which the IL-1β -IL-1R signaling pathway regulates skin inflammation remain unclear. Genetic studies have shown that IL-1β gene polymorphism can be used to distinguish early-onset and late-onset psoriasis. In our study, IL-1β protein level was also shown to be correlated with the treatment response of the disease. These studies suggest that IL-1B is closely related to the effect of guselkumab in the treatment of patients with psoriasis and is expected to be a prognostic target.

The first cells to respond to the pathogenesis of psoriasis are keratinocytes. When they are activated, these cells will secrete basic innate immune mediators. Overexpression of proinflammatory mediators like cytokines and chemokines cause to inflammatory. The major inflammatory cytokine IL-1β promotes the production of other mediators such as CXCL8. CXCL8 is an effective chemoattractant for neutrophils and is also thought to stimulate keratinocyte proliferation. CXCL8 levels in the serum of patients in psoriasis were reported by Zhen (20). In our study, we found that fast responders with psoriasis was mainly involved in low-density neutrophils, speculated that this may be caused by CXCL8 reduction.

S100A12 (a member of the S100 proteins family) can be actively secreted by acti-vated granulocytes and having potent chemotactic activity (21). S100A12 was considered the most promising active marker for psoriasis in the Wilsmann-Theis study. Blood-brain barrier studies have shown that neutrophil release of matrix metallopeptidase 9 (MMP-9) leads to vascular barrier dysfunction and hyperpermeability by disrupting tight junctions and cytoskeletal integrity. In psoriasis, vascular remodeling of the affected skin is critical for maintaining nutrient supply and inflammatory cell accumulation in the hypertrophic epidermis. Chen’s data show that neutrophils and MMP-9 regulate vascular remodeling in psoriasis by regulating VEC function in the skin, which provides a powerful strategy for the treatment of psoriasis (19). In our analysis, S100A12 also plays an important role, and we will conduct further studies on this cytokine in order to draw stronger conclusions.

## 5 Conclusion

In conclusion, in our study, we demonstrated the efficacy of guselkumab in the treatment of psoriasis and found that some key inflammatory factors were down-regulated at an early stage in patients with fast responders, suggesting that the levels of inflammation in these patients may be well suppressed at an early stage. This suggests that the detection of key inflammatory factors in patients at the early stage of clinical practice can predict the reactivity of patients to guselkumab and play a guiding role in the later treatment.

## Data Availability Statement

The datasets presented in this study can be found in online repositories. The names of the repository/repositories and accession number(s) can be found below: NCBI; PRJNA810783. GEO; GSE201397.

## Ethics Statement

The study was approved by the Ethics Committee of dermatology Hospital affiliated to Tongji University. The patients/participants provided their written informed consent to participate in this study.

## Author contributions

Conception and design: JL, YW, NY, YS. Provision of study materials: JL, YW, YL, XZ. Collection and assembly of data: YG, YD. Data analysis and interpretation: JL, YW, NY, YS. Manuscript writing: All authors. Final approval of manuscript: All authors. All authors contributed to the article and approved the submitted version.

## Funding

This work was sponsored by grants from National Natural Science Foundation of China (No.81872522, 82073429), Youth Program of National Natural Science Foundation of China (No.82003335, 81803120). Innovation Program of Shanghai Municipal Education Commission (No.2019-01-07-00-07-E00046), the Program of Science and Excellent Subject Leader Program of Shanghai Municipal Commission of Health and Family Planning (No. 2018BR30), Technology Commission of Shanghai Municipality (No. 18140901800), Clinical Research Program of Shanghai Hospital Development Center (No. SHDC2020CR1014B, SHDC12018X06), Program of Shanghai Academic Research Leader (No. 20XD1403300) and The National Key Research and Development Program of China (no. 2018YFC1705301, 2018YFC1705305), Clinical training program (lcfy2020-02).

## Conflict of Interest

The authors declare that the research was conducted in the absence of any commercial or financial relationships that could be construed as a potential conflict of interest.

## Publisher’s Note

All claims expressed in this article are solely those of the authors and do not necessarily represent those of their affiliated organizations, or those of the publisher, the editors and the reviewers. Any product that may be evaluated in this article, or claim that may be made by its manufacturer, is not guaranteed or endorsed by the publisher.
